# TMS-EEG and resting-state EEG applied to altered states of consciousness: oscillations, complexity, and phenomenology

**DOI:** 10.1016/j.isci.2023.106589

**Published:** 2023-04-07

**Authors:** Andres Ort, John W. Smallridge, Simone Sarasso, Silvia Casarotto, Robin von Rotz, Andrea Casanova, Erich Seifritz, Katrin H. Preller, Giulio Tononi, Franz X. Vollenweider

**Affiliations:** 1 Neurophenomenology of Consciousness Lab, Department of Psychiatry, Psychotherapy and Psychosomatics, Psychiatric Hospital, University of Zurich, Zurich, Switzerland; 2 Department of Biomedical and Clinical Sciences, University of Milan, Milan, Italy; 3 IRCCS Fondazione Don Carlo Gnocchi Milano, Milan, Italy; 4 Department of Psychiatry, Psychotherapy and Psychosomatics, Psychiatric Hospital, University of Zurich, Zurich, Switzerland; 5 Department of Psychiatry, University of Wisconsin, Madison, WI, USA

**Keywords:** Medical imaging, Pharmacology, Clinical neuroscience

## Abstract

Exploring the neurobiology of the profound changes in consciousness induced by classical psychedelic drugs may require novel neuroimaging methods. Serotonergic psychedelic drugs such as psilocybin produce states of increased sensory-emotional awareness and arousal, accompanied by increased spontaneous electroencephalographic (EEG) signal diversity. By directly stimulating cortical tissue, the altered dynamics and propagation of the evoked EEG activity can reveal drug-induced changes in the overall brain state. We combine Transcranial Magnetic Stimulation (TMS) and EEG to reveal that psilocybin produces a state of increased chaotic brain activity which is not a result of altered complexity in the underlying causal interactions between brain regions. We also map the regional effects of psilocybin on TMS-evoked activity and identify changes in frontal brain structures that may be associated with the phenomenology of psychedelic experiences.

## Highlights

• Psilocybin induces a state of increased sensory-emotional awareness and arousal
• Psilocybin induces both spontaneous and TMS-evoked EEG spectral changes
• Perturbational complexity is unaltered unlike that of spontaneous EEG activity
• These results help characterizing drug-induced altered states of consciousness

## Graphical abstract

**Figure:**
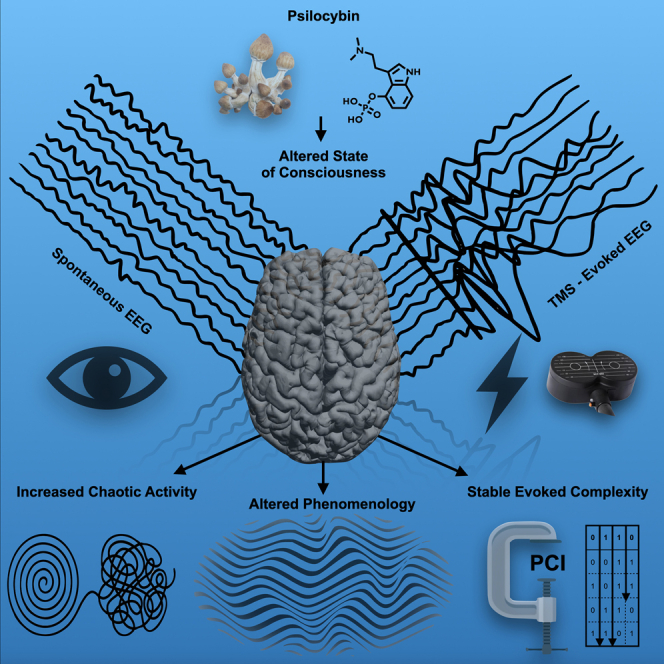

## Introduction

Altered states of consciousness (ASCs) induced by psychedelic drugs represent an interesting tool for investigating the neurobiological basis of conscious experience. These are states of altered mood, sensations, modes of thought, self-awareness, and the perceptual boundaries between them.^1^ ASCs can be induced by administering compounds such as psilocybin, lysergic acid diethylamide (LSD), and N, N-dimethyltryptamine (DMT)^2^^,^^3^^,^^4^ and it is thought that these effects are mediated primarily by serotonin 2A receptor (5HT-2AR) agonism.^2^^,^^3^^,^^4^^,^^5^^,^^6^^,^^7^^,^^8^^,^^9^ There continues to be growing interest in the potential for these substances as psychotherapeutic interventions for conditions such as affective and addictive disorders.^10^^,^^11^^,^^12^ Therefore, models of their neurophysiological effects should be refined in tandem with the development of treatment models.

The presence of a conscious experience is typically associated with behavioral responsiveness and entails differing degrees of awareness and arousal—both of which are high during waking and low during non-rapid eye movement (NREM) sleep and anesthesia. Aside from the pathological dissociation exhibited by neurological patients affected by disorders of consciousness, an even more striking case is represented by the presence of consciousness in the form of dreaming during rapid eye movement (REM) sleep,^13^ a state characterized by low arousal and behavioral unresponsiveness. In this context, compared to typical waking consciousness, 5HT-2AR agonists elicit experiences of heightened arousal and sensory as well as emotional awareness.^12^ Due to their shared phenomenology, these ASCs have been described as waking lucid dream states.^8^^,^^14^

Empirical work has revealed that the diversity of electroencephalographic (EEG) signal patterns is high whenever consciousness is present regardless of the degree of behavioral responsiveness and arousal, to an extent that is comparable to that obtained during full-fledged wakefulness.^15^^,^^16^^,^^17^^,^^18^ Interestingly, psychedelic-induced ASCs are accompanied by increased EEG signal diversity relative to typical wakefulness and dreaming.^19^^,^^20^^,^^21^ This increased signal diversity could be due to enhanced causal interactions within the brain, or rather be a product of unstructured and chaotic neuronal activity.^21^^,^^22^

To address this unknown, a potentially useful strategy is to assess the response of the brain to direct perturbations.^22^ Due to its non-invasiveness, Transcranial Magnetic Stimulation (TMS) is particularly suited to this aim, and the analysis of the ensuing EEG response can reveal how activity propagates in space and time. The complexity of these spatiotemporal activity patterns is then measured by the Perturbational Complexity Index (PCI) which, just as for spontaneous EEG signal diversity, displays high and similarly distributed values for both dreaming and waking states.^23^^,^^24^^,^^25^

Measuring the complexity ensuing from direct cortical perturbations rather than that of spontaneous brain activity is both theoretically^26^ and methodologically^27^ relevant as it tackles the problem of measuring causal relationships as opposed to statistical dependencies. This contrasts the influence of spurious sources of integration, such as common drivers and correlated inputs, and minimizes the influence of noise, which can artificially affect complexity estimations, e.g., in the presence of random patterns. As such, this comparison would help distinguish between a unitary system made of tightly interacting elements (the putative ideal candidate substrate for consciousness) and an aggregate of largely independent generators of activity.

As yet, only one study has explored the behavior of PCI and spontaneous EEG signal diversity during ASCs induced by sub-anesthetic doses of the N-methyl-D-aspartate receptor (NMDAR) antagonist ketamine.^21^ The ASC induced by ketamine is characterized by personal dissociation and disembodiment.^19^^,^^21^ Interestingly, results showed stable PCI values despite increased spontaneous signal diversity. From this, the authors concluded that ketamine-induced ASCs may be states of increased chaotic neuronal activity, but unaltered in the complexity of the underlying causal interactions.

Here, we sought to understand whether these effects are ketamine-specific or rather reproducible for classical psychedelics using the 5HT-2AR agonist psilocybin. Specifically, we analyzed spontaneous EEG signal diversity as well as PCI values and employed a placebo-randomized double-blind study on a larger sample size with respect to^21^. We were able to verify several findings of earlier studies and explored regional drug effects along the rostro-caudal axis. We highlight an important distinction to be made between spontaneous and evoked brain activity during psychedelic-induced ASCs. We demonstrate that, despite preserving similar PCI values, TMS-evoked activity is significantly altered by psilocybin and provide the first evidence that these effects are associated with ASC phenomenology.

## Results

**Figure 1:**
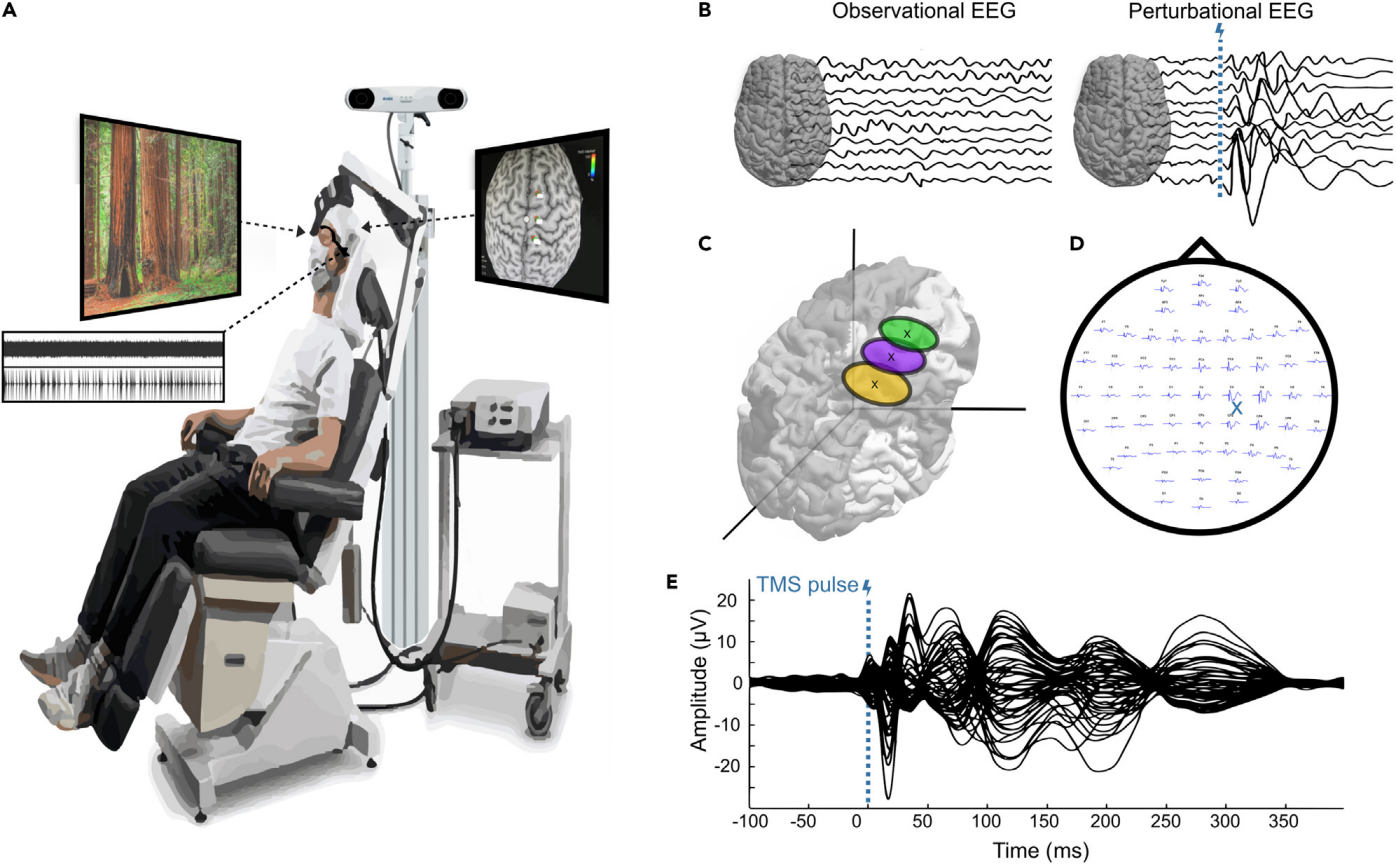
The TMS-EEG setup for real-time monitoring (A) TMS-EEG apparatus comprising a movable chair, 64-channel high-density EEG cap, stimulator, foam-covered coil, and neuro-navigational device. Neuro-navigation integrates individual anatomy from T1 MRT recordings via sensor-mounted glasses on the participant’s head and a sensor-mounted coil. Slow-moving natural scenery was shown to participants to ensure overall comfort and attention throughout. Individually titrated white noise in combination with randomized recordings of the TMS discharge click sounds were played through noise-canceling earphones to suppress auditory evoked potentials (AEPs). (B) Two data acquisition approaches: conventional spontaneous EEG as an ‘observational’ recording method and a ‘perturbational’ method via local cortical stimulations to assess effective neural interactions. (C) Stimulation areas of interest were the premotor cortex (BA 06, green), primary motor cortex (BA 04, purple), and primary somatosensory cortex (BA 1–3, yellow). Each area was stimulated with 200 trials per recording session. (D) Online evaluation of TMS-evoked-potential (TEP) size and effectiveness of stimulation was performed on a separate computer before and during recordings (here is shown an example of a somatosensory stimulation by a blue cross). TEPs for individual electrodes are displayed topographically for a −100 to 400 ms time window with respect to the TMS-pulse. (E) Butterfly plot of the trial average TEP of overlayed individual electrodes.

Participants underwent two randomized double-blind recording days with a two-week interval, wherein either psilocybin or placebo capsules were administered. All participants (N = 22) experienced both drug conditions and separate same-day EEG and TMS-EEG recording periods, followed by psychometric questionnaires. Following drug administration, an EEG recording of restful eyes open (EO), and eyes closed (EC) conditions was performed, which was then followed by three separate TMS-EEG recording sessions with TMS targeted to three cortical areas along the rostro-caudal axis: Premotor Cortex (PM), Primary Motor Cortex (M1), and Primary Somatosensory Cortex (S1) (Figure 1).

### Contrasting effects of psilocybin on spontaneous and perturbational complexity

Measures of EEG signal complexity were applied to TMS-EEG and spontaneous EEG as defined by the Perturbational Complexity Index (PCI) and spontaneous Lempel-Ziv complexity (LZc), respectively. PCI reflects the number of unique binary sequences of significant spatiotemporal activity at the source level with respect to the pre-TMS baseline activity. On the other hand, LZc measures the number of unique binary sequences of significant events in the resting-state EEG at the sensor level relative to a statistically defined threshold.

### Perturbational complexity index is stable and unaltered by psilocybin

**Figure 2:**
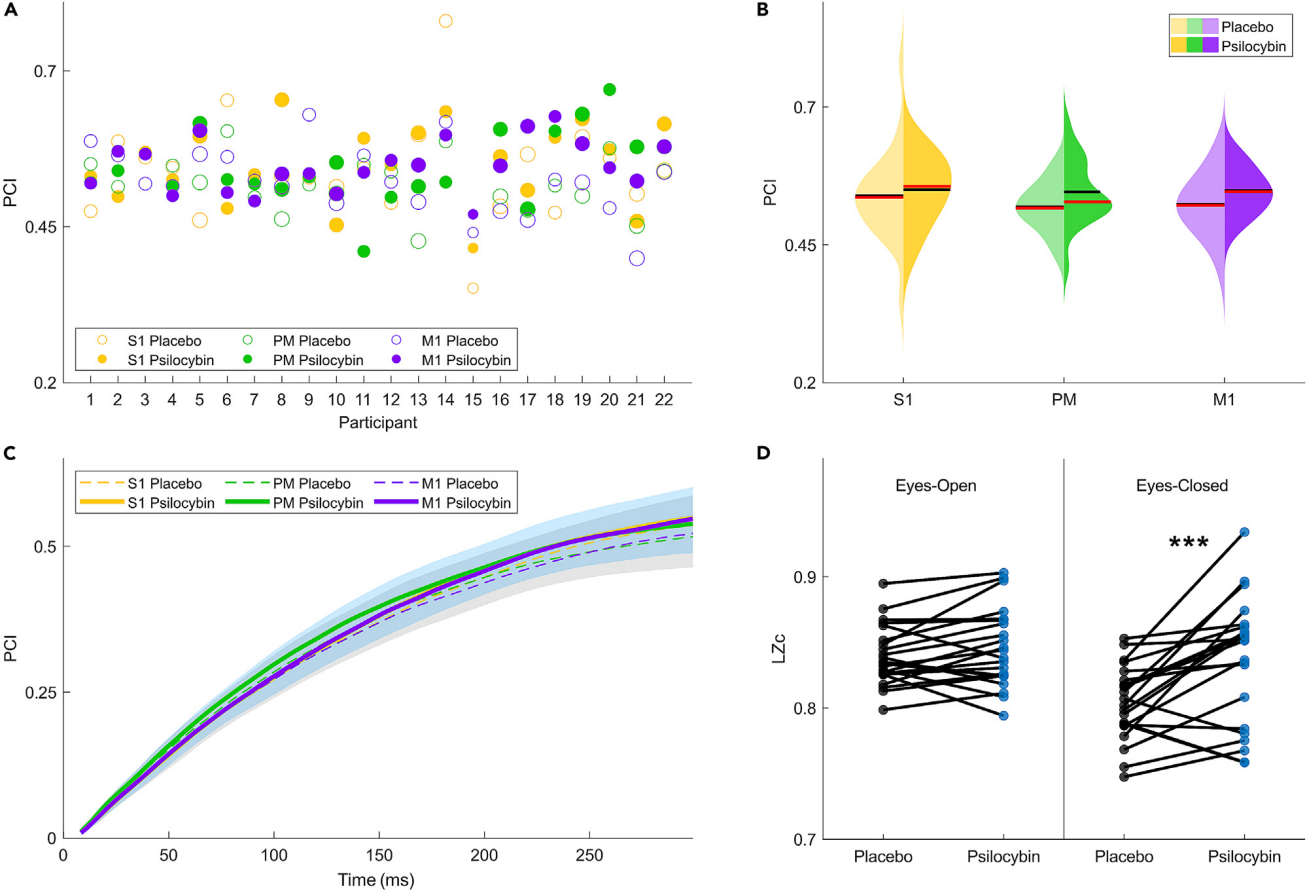
Psilocybin-induced brain states sustain perturbational complexity with altered spontaneous signal complexity (A) PCI values for all participants, color-coded by stimulation site and drug conditions, marker sizes proportional to the psilocybin dose (15 or 20 mg). (B) Split-violin plot of PCI values grouped by stimulation site. Placebo measures are lightly shaded and psilocybin measures are heavily shaded on each violin. The mean (black line) and median (red line) are plotted on each distribution. (C) The value of PCI at each time point (8–300 ms) was computed and finds no change in its temporal evolution (Figure S1). The shaded regions represent the range of placebo values (gray) and psilocybin values (blue) for all stimulation sites. (D) Normalized Lempel-Ziv complexity (LZc) of spontaneous EEG recordings. Each line represents the change in value between drug conditions for an individual. S1: Primary Somatosensory Cortex (yellow); PM: Premotor Cortex (green); M1: Primary Motor Cortex (purple) (Table S1). Asterisks denote statistical significance (∗p < 0.05, ∗∗p < 0.01, ∗∗∗p < 0.001).

Individual PCI values across stimulation sites for on- and off-drug conditions appear to be similarly distributed and within the range of those previously published^23^^,^^24^^,^^28^ (Figures 2A and 2B). The distribution of PCI values was not statistically significantly changed—albeit showing increased mean and median values—after the administration of psilocybin as compared to placebo for any of the stimulated regions along the rostro-caudal axis (Table S1). Pairwise t-testing reveals no significant changes in PCI values and one-way ANOVA applied to pairwise PCI change (psilocybin minus placebo) also finds no significant differences between conditions and groups (SS = 0.003, df = 2, MS = 0.002, F = 0.31, Prob>F = 0.73). To account for the possibility that this tendency may result from a change in the evolution of PCI following the TMS pulse, we calculated PCI per millisecond (PCI(t)). PCI(t) was demonstrated to grow at a comparable rate in both placebo and psilocybin conditions (Figure 2C). Repeated measures ANOVA was applied to PCI values as 50 ms interval bins for each stimulation site to assess within-subject effects of conditions and times but found no significant interactions (all of p > 0.1) (Figure S1). TMS-evoked complexity was also measured directly from channel activity, i.e., without source modeling, using the computationally faster alternative—PCI-State Transitions (PCI-ST).^25^ Consistent with PCI measured at the source level, psilocybin did not significantly alter PCI-ST (Figure S2).

### Psilocybin-induced brain states display increased electroencephalographic signal diversity at rest

We next computed LZc of the spontaneous EEG activity to assess the ongoing signal diversity present at rest. Compared to placebo, psilocybin significantly increased LZc with eyes closed (p < 10^-3^) but not eyes open (Figures 2D and S3). Interestingly, closing the eyes significantly reduced LZc during the placebo condition (p < 10^-4^)—an effect that was not present after the administration of psilocybin (p > 0.1). The LZ complexity of single channels (LZs) was also computed to provide additional information about the spatial distribution of these changes in LZc (Figure S4). After correcting for the false discovery rate (FDR), psilocybin significantly increased LZs at posterior-occipital and medial-frontal channels during the eyes closed condition, as well as the eyes open condition but for fewer channels. To investigate the effect of periodic signals, LZs values were normalized with respect to a phase-shuffled version of the resting-state EEG activity (LZsN), which removes continuous periodicity while retaining spectral power per frequency. Significantly affected regions were reduced to a few sparsely distributed channels with lower T-statistic values.

### Psilocybin induces distinct spectral changes in electroencephalographic and transcranial magnetic stimulation-electroencephalographic activity

**Figure 3:**
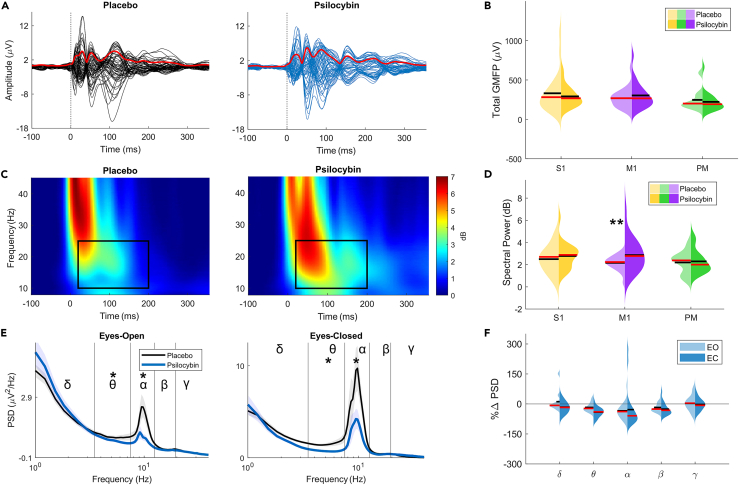
Psilocybin-induced changes in the TMS-evoked potential (A) An example of the TMS-evoked response (Participant P13) to primary motor cortex (M1) stimulation. Channel potentials are plotted over time and overlayed to visualize the TMS-evoked Potential (TEP) for placebo (left, black) and psilocybin (right, blue) conditions. Global Mean Field Power (GMFP) is overlayed in red. (B) The total GMFP value (50–200 ms) of the TEP for each stimulation site. GMFP values were baseline-corrected with respect to the mean value 100 ms pre-stimulus. (C) The Event-Related Spectral Perturbation (ERSP) response for the TEPs is plotted in (A). The rectangular box highlights a time-frequency space of empirical interest (20–200 ms, 10–25 Hz). (D) Mean values for the time-frequency space highlighted in (C) which comprises 10–25 Hz power in the first 20–200 ms following the TMS pulse. Psilocybin only significantly increased the M1 cortex spectral response power (p < 0.01). (E) Channel-average spontaneous EEG Power Spectral Density (PSD) on a semi-log scale. Shaded areas represent the standard deviation of placebo (gray) and psilocybin (blue) curves and solid line curves represent the average across participants. (F) Violin plot of the percentage change in mean frequency band power between drug conditions (Psilocybin – Placebo). Vertical lines divide the bands used for analysis: Delta (δ, 1–4 Hz); Theta (θ, 4–8 Hz); Alpha (α, 8–12 Hz); Beta (β, 12.5–20 Hz); Gamma (γ 20–40 Hz). Eyes open (EO, left) and Eyes closed (EC, right) recordings were analyzed separately. Theta- and Alpha-band powers are significantly changed in the psilocybin condition relative to the placebo (Table S1). Asterisks denote statistical significance (∗p < 0.05, ∗∗p < 0.01, ∗∗∗p < 0.001).

We next sought to verify that the stability of PCI on- and off-psilocybin was not trivially explained by unaltered TMS-evoked responses. To account for known differences in evoked response properties across cortical sites,^29^ we probed the effect of psilocybin along the rostro-caudal axis. Individual TMS-evoked potentials (TEPs) were visualized by overlaying the trial-averaged voltage of each scalp electrode (Figure 3A). The variation in instantaneous voltages across the scalp was quantified using Global Mean Field Power (GMFP). Psilocybin-induced spectral changes were assessed for EEG and TMS-EEG activity using Power Spectral Density (PSD) and global Event-Related Spectral Perturbation (ERSP), respectively.

### Cortical reactivity is not altered by psilocybin

To summarize the complex structure of TEPs at each stimulation site, we first described the global cortical reactivity on- and off-drug using GMFP. Total GMFP was calculated as the sum of time-resolved GMFP values between 50 and 200 ms post-stimulation. No statistically significant changes in total GMFP were observed after the administration of psilocybin at any of the stimulation sites (Figure 3B and Table S1). We evaluated the time-resolved GMFP changes induced by psilocybin which identified sparse clusters of statistical significance (after correcting for multiple comparisons) post-stimulus for the M1 and Premotor cortex (Figure S5).

### Transcranial magnetic stimulation-related spectral activity is increased by psilocybin

TMS-evoked responses were transformed into the time-frequency domain to reveal non-stationary spectral changes induced by psilocybin. Interestingly, individuals often varied in the effect of psilocybin on their TEPs across all three stimulation sites. By averaging the time-frequency structure over all channels, the global ERSP was used to further summarize drug-specific effects on TMS-related spectral activity. The mean difference in ERSP between drug conditions suggested that psilocybin had affected spectral power for all stimulation sites. This increase was greatest for the initial 20–200 ms and within a frequency window of 10–25 Hz (Figure S6). Mean global ERSP power in this time-frequency window was found to significantly increase during M1 stimulation for psilocybin compared to placebo (Figures 3C and 3D and Table S1).

### Psilocybin reduces resting electroencephalographic spectral power

We next sought to compare the TMS-related spectral changes to broad spectral changes which also appear while at rest, i.e., without stimulation. Resting-state PSD was computed for EO and EC conditions and segmented into conventional EEG bands for analysis. For both EO and EC recording conditions, both mean Theta (4–8 Hz) and Alpha (8–12 Hz) power were significantly decreased after psilocybin administration (Figure 3E and Table S1).

### Exploring associations between phenomenology and neurophysiology

Psychometric questionnaires were used to assess the phenomenological experiences of each participant by measuring the degree of qualitative dimensions of the psilocybin-induced ASC directly after psychoactive effects had ceased. The 11-Dimensional Altered States of Consciousness (11D-ASC) rating scale was used to test for relationships between phenomenological, EEG, and TMS-EEG features.^30^ EEG and TMS-EEG measures were converted to relative change values (with respect to placebo, see STAR Methods) to correct for individual baseline neurophysiological states.

### Psilocybin induced strong and varied alterations in phenomenology

**Figure 4:**
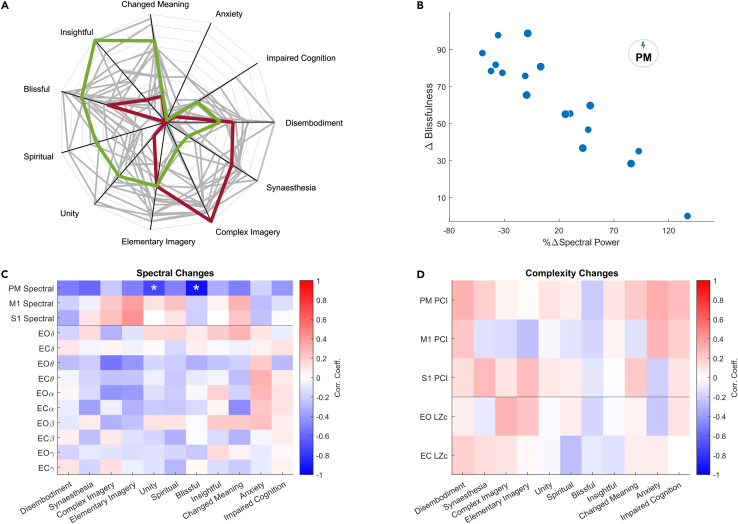
Phenomenological changes are reflected in altered TMS-related spectral changes (A) Radar plot of the change in 11D-ASC scores from placebo to psilocybin recordings. Individual 11D-ASC ratings are plotted in gray to demonstrate the diversity of phenomenological ratings. Individual participant scores P7 (red) and P13 (green) are colored to highlight the individuality of psilocybin-induced experiences during this study. Axes are from 0 to 100% of the maximum score with bins of size 10. (B) Change in the 11D score ‘Blissful’ relative to placebo plotted against percentage relative change in mean 10–25 Hz power (20–200 ms window following the TMS pulse) induced by psilocybin when stimulating PM cortex. Marker sizes are proportional to the psilocybin dose (15 or 20 mg). (C) Correlation matrix of Pearson correlation coefficient values for change in 11D-ASC scores and relative change in spectral spontaneous EEG and TMS-EEG metrics. TMS-EEG and EEG rows are separated by a horizontal line. Asterisks indicate significant correlations (|R| > 0.6, p < 0.05). (D) Identical procedure as in (C) for EEG and TMS-EEG complexity measures. S1: Primary Somatosensory Cortex; PM: Premotor Cortex; M1: Primary Motor Cortex; LZc = Normalized Lempel-Ziv complexity; Eyes open (EO), Eyes closed (EC).

Ten of the eleven dimensions of the 11D-ASC scales were significantly increased by the application of psilocybin (Figure 4A). Elementary Imagery, Complex Imagery, AudioVisual Synesthesia, Disembodiment, Changed Meaning of Percepts, Cognitive Impairment, Unity, Insightfulness, Blissfulness, and Spiritual experience were all significantly increased by the application of psilocybin (Table S2). Only Anxiety was not significantly affected after correcting for multiple comparisons (Bonferroni correction for n = 11 comparisons). Significant phenomenological changes as assessed by the 11D-ASC scales demonstrate that sufficient doses were provided to elicit the desired subjective alterations.

### Relative change in transcranial magnetic stimulation-related 10–25 Hz power correlates with phenomenology

A subset of features of the TMS-evoked response during psilocybin-induced brain states was found to correlate with phenomenological effects. The strongest correlation coefficient reported here (Figure 4B) is the change of Blissfulness as a function of relative change in 10–25 Hz ERSP power (R = −0.924, p < 10^-6^) after TMS of the PM cortex. When comparing the relative change in TMS-related 10–25 Hz spectral power across stimulation sites with a relative change of resting-state EEG mean band power, we find evidence that the perturbational approach tends to yield more robust neurophenomenological correlates (Figure 4C). Relative change in spectral power was also correlated with an experience of Unity (N = 17, R = −0.7, p = 0.001). We also determined that Blissfulness was strongly correlated with other 11D-ASC sub-dimensions such as Unity, and therefore the other correlations found here with TMS-evoked PM cortex spectral changes are likely to be due to multicollinearity (Figure S7). Neither evoked nor spontaneous relative change of complexity measures across stimulation sites and behavioral states, i.e., EO or EC, provided absolute Pearson correlation coefficients of 0.5 or greater for 11D-ASC scores (Figure 4D).

## Discussion

We report that the TMS-evoked response is significantly altered but no more or less complex during a psilocybin-induced ASC compared to typical wakefulness. This stands in contrast to the increased spontaneous signal diversity observed at rest with eyes closed. Through attempts to understand how these two orthogonal measures of complexity are generated and related together, we found distinct properties of ongoing vs. evoked rhythms during 5HT-2AR-mediated psychedelic states. This perturbational approach provided further insights as to the potential relevance of brain (particularly frontal regions) state changes during psychedelic experiences.

### Explaining the increased spontaneous but not evoked signal diversity

The contrast between the increased spontaneous signal diversity and the unaltered TMS-evoked spatiotemporal complexity is intriguing but not surprising. The spontaneous signal diversity measured by LZc reflects the differentiability of EEG patterns over time but assumes spatial interactions are present without explicitly accounting for them.^31^ This increased temporal differentiability could be due to spatially separate brain regions, which may normally share common driver inputs, transitioning to a state of increased independence from one another. EEG activity following direct cortical stimulation, however, reveals the spatiotemporal structure of causal interactions in the brain, thus explicitly also estimating the degree of integration. The absence of a change in PCI during an ASC, yet the increase in LZc, suggests that the underlying brain state is unaltered in its ability to maintain complex causal interactions while allowing ongoing activity to be more chaotic or noisy. Critically, this result for a 5HT-2AR-mediated ASC is consistent with the only other study of PCI and LZc for sub-anesthetic doses of ketamine.^21^ This strongly suggests that these effects occur for both classical psychedelic compounds and ketamine administration, despite differing receptor targets of action.

We find that reducing visual input by closing the eyes decreased LZc during the placebo condition. For the psilocybin condition, however, participants often report elementary and complex visual experiences. Consistent with previous work,^19^ we find that LZc is higher after psilocybin administration compared to placebo when the eyes are closed despite the lack of exogenous visual stimuli. It is plausible that this heightened signal diversity reflects endogenously generated chaotic neuronal activity during ASCs. However, we found no correlations between the degree of change in LZc and the reported visual effects of psilocybin. Eye closure and therefore reduced visual processing is known to result in increased spectral power, especially within the alpha frequency band. It has often been speculated that periods of highly synchronized alpha-band power introduce repetitive patterns in EEG signals which reduce pattern diversity and therefore LZc.^20^^,^^21^^,^^22^^,^^31^ As we had observed this typical reduction in alpha-band power, we explored how this related to our LZc findings. We found that changes in both LZc and alpha-band power were significantly negatively correlated in both placebo and psilocybin conditions (Figure S8). By performing phase-shuffle normalization of LZc (LZcN) we could partially account for periods of high oscillatory behavior. LZcN was uncorrelated with alpha-band power, which predominantly affected signal diversity when alpha-band power was high. Given that alpha-band power is often lower in the psilocybin condition, it is plausible that increased LZc values are a result of fewer or reduced alpha-band oscillatory sequences. Resting-state EEG alpha-band activity is characteristically strongest on posterior channels,^32^ while theta-band activity is present in the medial frontal channels.^33^ Given that we find evidence for these two frequency bands being reduced by psilocybin, it is plausible that decreased spectral power would also be accompanied by increased LZ values at these locations. We present single-channel LZ (LZs) data for this purpose, which suggest that LZs is increased on medial frontal and posterior-occipital channels for both eyes open and eyes closed conditions, the effect of which is diminished when LZs is normalized by phase-shuffling (LZsN) (Figure S4). We speculate that there is evidence to suggest an influence of spectral changes induced by psilocybin on spontaneous EEG signal diversity.

Despite reduced spectral power around 10Hz in spontaneous EEG activity, oscillatory activity in similar frequencies could be amplified by perturbation (Figures 3C and 3D). The loss of spontaneous oscillatory power may reflect a diminished capacity for neurons to synchronize at these frequencies. Although, the TMS-evoked responses suggest that neuronal populations can still sustain synchronized entrainment when perturbed. It is important to note, however, that the ongoing brain rhythms at rest may be the result of global network behavior,^34^ such as the default mode network (DMN)^35^ and dorsal attention network,^36^^,^^37^ whereas evoked rhythms should reflect dynamics following the perturbation of a specific part of a network of interacting brain regions.^21^^,^^22^^,^^29^ Due to this orthogonality between observational and perturbational approaches to characterizing drug-induced changes in brain state, purely observational brain activity may not necessarily predict how a brain may react to external stimuli. For this reason, previously speculated functional roles of specific frequency bands in resting EEG activity during ASCs^38^ may be clarified by employing a multimodal approach.^7^

### The role of the frontal cortex and phenomenological altered states of consciousness correlates

To further explore the psilocybin-induced changes in TMS-related oscillations we explored their regional aspects. Probing stimulation sites along the rostro-caudal axis revealed several evoked response properties which were altered by psilocybin across frontal structures. Increased 10–25 Hz ERSP power when stimulating the M1 cortex was noticed following psilocybin administration. We also clarified that psilocybin does not significantly alter the overall GMFP during the early TMS-evoked response at any stimulate site, which reflects no global change in reactivity across the scalp. Performing a time-resolved GMFP analysis found that psilocybin altered a sparsely distributed set of time points. However, given the high variation across individuals and that these are not believed to be due to any specific TEP component, we propose that psilocybin does not reliably alter global reactivity across participants. Psilocybin also caused varied 10–25 Hz ERSP power changes across individuals which were strongly correlated with changes in experienced Blissfulness and Unity, as measured by the 11D-ASC scales. However, similarly strong correlations were not observed for changes in spontaneous EEG frequency band power. In a single-subject study, it was reported that for a meditation expert, shifting between phenomenologically distinct meditation states was accompanied by changes in PM cortex 25–35 Hz ERSP power.^39^

The reaction of the frontal structures and their relationship to phenomenology could be a result of their position within the cortical hierarchy;^40^^,^^41^ frontal structures are typically associated with executive function and behavioral control.^42^^,^^43^ It is plausible that frontal cortical structures are disproportionately influenced by psilocybin^4^ and by the distribution of 5-HT2ARs present there.^44^ These results offer further evidence for the hypothesis that classical psychedelic drugs which target primarily 5HT-2ARs affect frontal brain structures via altered thalamocortical dynamics.^9^^,^^12^ While the receptor mechanisms for ketamine and psilocybin may be different, the “glutamatergic overspill” hypothesis posits that both ketamine and serotonergic hallucinogens have a shared neuronal mechanism; both sets of compounds modulate medial prefrontal cortex (mPFC) Layer 1 (L1) and 5 (L5) pyramidal neurons and/or activation of metabotropic glutamate type 2 (mGlu2) autoreceptors on thalamocortical afferents to mPFC. This creates a positive feedback loop of glutamate release in populations of neurons in the frontal cortex which reduces the firing threshold and increases spontaneous firing. These effects have been further implicated in the hallucinogen-induced head-twitch response in mice, motoric impulsivity, and anti-depressant effects in rats.^45^ This model could plausibly account for both the multiple instances of ASC-relevant frontal region changes we have found and the increased spontaneous signal diversity which we verified in a hallucinogen acting primarily via another receptor target. However, the transferability of these rodent study results to models of human behavior is yet to be fully established.

### Conclusion

We have established that changes in spontaneous EEG signal diversity and stability of perturbational complexity are reproducible across psychedelic-induced ASCs acting via different primary receptor types. We provide further evidence that PCI reliably indicates which brain states are accompanied by awareness but is not a measure for specific features of phenomenology. By verifying LZc as a biomarker for psychedelic-induced ASCs, we found that increases in spontaneous signal diversity are potentially a product of previously known spontaneous EEG spectral changes induced by psilocybin. We provide evidence for psilocybin-induced losses in spontaneous rhythmicity which can be elicited and amplified when perturbed by TMS, suggesting that this perturbational neuroimaging approach provides insights for psychedelic-induced ASCs which are distinct from resting-state EEG. Regional drug effects on TMS-evoked rhythms and their spatiotemporal structure were shown to be associated with specific psilocybin-induced ASC phenomenological states.

### Limitations of the study

While the sample size we used should be sufficient to investigate the effect of psilocybin on TMS-evoked EEG activity, the number of features that could be extracted was challenging for more complex analyses. Techniques for identifying latent feature spaces to better explore neurophenomenology often require larger sample sizes to detect measures of the greatest value reliably, and therefore it is hoped that larger studies could expand on the findings we report here. Although dose was not identified as a factor in our study for any neurophysiological metrics, expanding the dose range in future studies could prove insightful. Dose-per-weight values were chosen in this study to ensure that psilocybin did not elicit unwanted behaviors in participants, e.g., excessive movement, and retained meaningful comparisons between conditions. This study has also demonstrated that psilocybin can be applied safely in a TMS-EEG setting if performed appropriately. It is likely that uncontrolled yet known factors such as the “set and setting” (i.e., a participant’s expectations and their surroundings, respectively) could influence the quality of the psilocybin experience and subsequently the psychometric ratings.^46^^,^^47^^,^^48^^,^^49^ An example of this effect is hinted at by the low occurrence of anxiety reported in the 11D-ASC scores, which in the context of a TMS-EEG study reflects a balance between controlled recording conditions and the minimized discomfort of participants.

This is of course coupled with the challenge of effective double-blinding in research using psychedelic drugs.^50^ All steps were taken to maximally reproduce identical procedures on placebo and psilocybin recording days. It is also important to note that drug-induced ASCs are phenomenologically dynamic. Therefore, although TMS-EEG and EEG may capture brief periods of an ASC state they may not accurately reflect the ongoing transitions between phenomenological states experienced by participants. We also attempted to probe the higher visual cortex using TMS-EEG, however, practical constraints meant that these recordings were not guided by neuro-navigation and therefore were not included in the findings of this study (Figure S9). Theoretically, it may be also challenging to accurately discriminate the multidimensional changes in emotion, thought, perception, and self of ASCs using one-dimensional scales such as PCI and LZc^51^^,^^52^ which, by constitution may be quite sensitive but rather unspecific to the presence of any of the phenomenological dimensions. Future studies may therefore benefit from a multimodal approach as we have found it useful in this investigation of multiple brain regions, imaging methods, and measures.

## STAR★Methods

### Key resources table

**Table:** 

REAGENT or RESOURCE	SOURCE	IDENTIFIER
**Software and algorithms**		

Matlab 2021a	https://ch.mathworks.com/products/matlab.html	RRID:SCR_001622
BrainVision Recorder	https://www.brainproducts.com/	RRID:SCR_016331
Visor2	https://www.ant-neuro.com/products/visor2	N/A

### Resource availability

#### Lead contact

Further information and requests for resources should be directed to and will be fulfilled by the Lead Contact, John Smallridge (john.smallridge@uzh.ch).

#### Materials availability

This study did not generate new unique reagents.

### Experimental model and subject details

25 healthy, right-handed participants (7 females, 18 males, mean age 24.44, range from 21 to 29 years) were recruited, screened for eligibility, and investigated twice at a 14-day interval with TMS-EEG in a randomized, within-subject, placebo-controlled, and double-blind setting. All subjects provided written informed consent. The experimental protocol was approved by the local ethics committee of Zurich (BASEC Nr.: 2018-01866; NCT03853577). The use of psilocybin was authorized by the Swiss Federal Office of Public Health.

All participants were screened beforehand for past and present health conditions to exclude incompatibilities with psilocybin usage and/or TMS procedure. This included psychiatric interviewing, an internist check-up, blood analysis, electrocardiogram (ECG), EEG, and cerebral magnetic resonance tomography (cMRT). Urinalysis was done on experimental days to exclude recreational substance use and pregnancy. Three participants were excluded from analysis due to an insufficient peak-to-peak amplitude (<10 μV) of the initial TMS-EEG response within the first 20 trials of the online measurement, following the protocols outlined previously.^53^

### Method details

#### Procedures

All experimental procedures were performed at the Psychiatric University Hospital in Zurich, Switzerland (Figure 1). Every participant received bodyweight-adjusted psilocybin formulated as capsules of 5 mg PO (20 mg over 80 kg, 15 mg for 50–80 kg, 10 mg below 50 kg; mean dosage 225 μg/kg/participant) on one measurement day and placebo (100% mannitol) on the other in a counterbalanced manner. Spontaneous EEG (5 min of eyes open followed by 5 min with eyes closed) was recorded before starting TMS-EEG sessions. Subjects were stimulated at three different cortical sites on the right hemisphere near to midline, targeting the premotor cortex (Brodmann area 6), the primary motor cortex (Brodmann area 4), and the primary somatosensory cortex (Brodmann area 1–3). These stimulation sites were selected to cover multiple positions along the rostro-caudal axis and to minimize TMS-evoked muscle artifacts (typically associated with cortical lateral aspect stimulation) during the recordings. On each measurement day, before substance intake, a first baseline measure was performed at selected stimulation sites. Each TMS-EEG recording session consisted of 200 TMS pulses per stimulation site at 100 ms-jittered 2000–2500 ms intervals (a duration of ∼7 min each) perfomed while subjects kept their eyes open. The stimulation target was selected using neuro-navigation and guided by estimate E-field until the TMS-EEG response had a peak-to-peak amplitude of 10μV within 20 trials – The operational criterion previously established.^53^ Stimulation parameters, i.e., TMS-coil angle, tilt, position, and intensity, were applied as necessary to establish this EEG threshold criterion before recording began.^24^^,^^53^ In a minority of placebo TMS-EEG recordings which were of insufficient quality relative to their baseline pre-intake recording, the baseline recording was used as the non-psilocybin reference recording (Figure S10). Any such re-referenced recordings were excluded from any correlation analyses.

After substance intake, a meditative atmosphere was installed by dimming light sources, showing nature scenes on a screen, and playing down-tempo music. These measures were taken to create a pleasant change in the participants' state of consciousness as induced by psilocybin. Spontaneous EEG was recorded 60 min after oral ingestion, which was then followed by TMS-EEG recordings using the same target regions in randomized order and adopting the same stimulation parameters (stimulation intensity, coil angle/tilt). In addition, participants' ECG was continuously monitored, and blood pressure was taken at regular intervals. Subjective dimensions of the ASC experience were measured using questionnaires at the end of the experiment.

#### TMS-EEG apparatus

TMS was performed using a 75 mm focal-bipulse butterfly coil (MCF-B65, MagVenture, Neurolite AG, Switzerland) driven by a mobile stimulator unit (MagPro R30, MagVenture, Neurolite AG, Switzerland). TMS pulses were triggered from a laptop using a trigger box (E.M.S. Sistemi Elettromedicali, Italy).

EEG measurements were recorded by using TMS-compatible 2x32-channel BrainAmp MR + amplifiers (Brain Products GmbH, Germany) attached to high-density 64-channel caps (BrainCap-TMS, EasyCap, Germany). The impedance of all electrodes was kept below 5kΩ, EEG signals were filtered DC-1000 Hz, sampled at 5000 Hz, and referenced to an electrode positioned above the nasion. Two sensors were used to record the electrooculogram with a diagonal montage. Using these experimental settings, the duration of the TMS pulse artifact was shorter than 5 ms.

#### Altered state consciousness

Psilocybin is a classic and safe hallucinogenic substance to induce a well-defined and transient altered state of waking consciousness. In healthy human participants, it is generally not associated with long-term perceptual, cognitive, or neurological dysfunctions. It unfolds its phenomenological characteristics by partially agonizing primarily the serotoninergic 5-HT2AR, which in turn induces dose-dependent psychological destabilization of self-other boundaries, emotional changes, sensory and time perception alterations, spiritual feelings of unity, and insightfulness with the external world.^54^ Psilocybin was given as a single moderate dose orally (mean 225 μg/kg/participant) with a peak effect 60–80 min after ingestion and fading out of symptoms 4 h later. Considerate guidance of participants and maintaining a safe environment throughout the daylong experiment ensured no unwanted or unpleasant effects (e.g., anxious ego dissolution). The lab was kept warm >21°C, a separate screen showed slow-moving nature scenery, low volume down-tempo music was played, and the front wall was furnished with a colorful tree tapestry.

The Altered States of Consciousness Questionnaire (ASC), a well-validated and one of the most widely applied self-rating scale with 94 visual analog items (0–100%), was used to reliably quantify the subjective drug effects (psilocybin, placebo).^55^^,^^56^^,^^57^ The scale comprises 5 main dimensions (factors) of ASC: oceanic (self) boundlessness; dreadful ego dissolution, visual restructuralization, auditory alterations, and vigilance reduction, which can be further characterized by 11 second-order scales: Experience of unity; spiritual experiences; blissfulness; insightfulness; disembodiment; impaired cognition and control; anxiety; elementary imagery; complex imagery; audio-visual synesthesia; and changed meaning of percepts.^30^ This latter set of predefined subdimensions was used for this study because it provided a broad yet reliable set of features for correlation analyses.

#### TMS-EEG pre-processing

Pre-processing was performed using functions extracted from EEGLAB-2014b^58^ and custom-written functions for TMS-induced artifact removal. For each EEG channel, signals in a −2 ms to +5 ms window around the TMS onset were replaced with mirroring of the signals from −9 ms to −2 ms to remove the TMS pulse artifact. A moving average filter was then applied. Slow voltage drifts were excluded by fitting a low-order polynomial to the data and then subtracting it (‘detrending’). Bad channel and trial rejection was carried out and data were re-referenced to the channel average. 1–45 Hz filtering was used with a 50 Hz line noise filter. Independent component analysis (ICA) was used to remove common EEG artifacts such as eye movements, cardiac interference, and muscle activity, and TMS artifacts such as the TMS decay and TMS-induced muscle twitch responses. The data was downsampled from 5000 Hz to 1000 Hz and bad channels were interpolated. These pre-processing steps, when averaged across trials and aligned to the TMS event, produce the TMS-evoked potential (TEP).

#### EEG pre-processing

Functions from EEGLAB-2014b^58^ were used to remove artifacts and noise from resting-state EEG recordings. Detrending and 1^st^-order high-pass filtering (0.01 Hz cut-off) were applied, followed by low- and high-pass Butterworth filtering of 0.5 and 40 Hz, respectively, and a 50 Hz notch filter. Segmentation into 2-second epochs was then used for bad channel removal and interpolation, which were then re-referenced to mean channel activity. Bad epoch rejection was performed before ICA to remove common behavioral artifacts similar to TMS-EEG components.

#### GMFP

As used in previous studies,^28^^,^^59^ GMFP was computed per millisecond as the standard deviation of potentials across scalp electrodes, with respect to the mean channel potential at that time point. The application of GMFP was therefore to summarize the global spatial structure of the TMS-evoked response. Let *X* be the trial average of the EEG data for channels (*C*) and time-point (*t*),

$$GMFP\left(t\right)=\sqrt{\frac{1}{C}\sum_{i=1}^{C}{\left(X_{i}\left(t\right)-X_{mean}\left(t\right)\right)}^{2}}$$

GMFP was baseline-corrected by subtracting the mean GMFP of the pre-stimulus period (−100 ms to −1 ms) from all GMFP(t) values. Total GMFP is calculated as the sum of GMFP(t) values within a specific time window.

#### ERSP

The time-frequency domain of TMS-evoked responses was analyzed using event-related spectral perturbation (ERSP). ERSP applies wavelet transformation (Morlet method, 3.5 cycles) and was performed using the public license toolbox EEGLAB.^58^ The ERSP values of single channels were averaged to yield the global ERSP (gERSP) for each TMS-EEG session. Mean spectral power was defined here as the average gERSP value in a time window of 20–200 ms and a frequency window of 10–25 Hz. The time window was selected to avoid artifactually inflated mean gERSP values and summarize robust spectral features of canonical TMS-evoked responses, as previously established.^29^ The mean change in ERSP between conditions was used to select the frequency band for which all stimulation sites were affected which was empirically decided to be 10–25 Hz.

#### PSD

To analyze resting-state EEG in the time-frequency domain, we separately calculated the Power Spectral Density (PSD) of the potentials for eyes open and closed periods. Restricted to 1–45 Hz and implemented using the *pwelch* function from the EEGLAB toolbox.^58^ Reported mean values are the average power of all frequencies within the conventional bands.

#### PCI

PCI was computed using identical procedures to those employed by Casali et al. (2013)^23^: source modeling using empirical Bayesian optimization of the Weighted Minimum Norm constraint was used to localize electromagnetic sources of scalp EEG potentials; non-parametric statistics (alpha = 0.01) across trials were applied to binarize the TMS-evoked response, which labels significant source amplitudes per unit time and source. After sorting sources by their total significant events, Lempel-Ziv complexity (C) was then computed to measure the number of unique binary strings present in the spatiotemporal response (L). C is then normalized by the binary entropy (H) of L (Figure S11), which yields the PCI value. PCI as a function of time can be computed using the same procedure but using the momentary C_t_ instead of the final value of C. We ascertained that all binary matrices for which we computed PCI generated an entropy value larger than 0.08, i.e., displayed a percentage of significant values greater than the rate of false positives of the statistics (1%).

An alternative measure of evoked complexity, PCI-ST,^25^ was applied at the sensor level. Eigenvectors generated from channel activity by principal component analysis were then ranked by their eigenvalue. The set of eigenvectors contributing 99% of the response variance was retained for further analysis. Recurrence Quantification Analysis was then applied to identify points of recurrence in the amplitude of each eigenvector as a measure of ‘state transitions’. The product of the number of eigenvectors and their respective total state transitions forms the PCI-ST value (see Comolatti et al., 2019^25^ for further information).

#### LZc

To describe resting-state EEG signal complexity we applied Lempel-Ziv complexity (LZc) as used in previous studies.^19^^,^^20^^,^^21^^,^^31^ Consecutive sequences of epochs, i.e., epoch sets without time points rejected during data cleaning, were concatenated into 10-s epochs for analysis. The analytic signal of single-channel EEG potentials is used for thresholding, which results in binary sequences for each channel. The binary values across all channels per time point are then concatenated to form a single binary sequence for all channels and times. Recordings were segmented into 10-s epochs and the LZc of each epoch was calculated. LZc has been demonstrated to be sensitive to decreasing epoch lengths,^31^ and therefore the epoch size was chosen to minimize this effect of insufficiently long epochs on LZc values. The LZc value for the recording is then reported as the average value across all epochs. To prevent occasional outlier epochs with excessively large or small values from skewing the average LZc, only values between the 25^th^ and 75^th^ percentile of a distribution of LZc values within each epoch were used.

### Quantification and statistical analysis

All statistical analyses were performed using built-in functions from either MATLAB or R-studio. Prior to null hypothesis testing using t-test functions all variables were checked using the Kolmogorov-Smirnov test to confirm their assumed normal distribution. Statistical analyses performed in MATLAB were reproduced using R-studio by different individuals who were blinded to the true drug conditions of the datasets. Statistical procedures which comprise analytical methods used in this work, e.g., ERSP and PCI, can be found in their respective publications.

### Additional resources

This paper did not create any additional resources.

## Data Availability

• Data reported in this paper may be shared by the lead contact upon request. • This paper does not report original code. • Any additional information required to reanalyze the data reported in this paper is available from the lead contact upon request.
