# Supplementary material for: TMS-EEG and resting-state EEG applied to altered states of consciousness: oscillations, complexity, and phenomenology
Source: iScience. 2023 Apr 7;26(5):106589. doi: 10.1016/j.isci.2023.106589 (PMC10149373; doi:10.1016/j.isci.2023.106589)
Supplement: Document S1. Figures S1–S11 and Tables S1 and S2 [file mmc1.pdf]

**iScience, Volume *26***

## Supplemental information

# TMS-EEG and resting-state EEG applied

# to altered states of consciousness:

# oscillations, complexity, and phenomenology

**Andres Ort, John W. Smallridge, Simone Sarasso, Silvia Casarotto, Robin von Rotz, Andrea Casanova, Erich Seifritz, Katrin H. Preller, Giulio Tononi, and Franz X. Vollenweider**

| | **Placebo** (mean, std) | **Psilocybin** (mean, std) | **T-value**, t(df) | **P-value** |
|---|---|---|---|---|
| PCI (S1) | 0.54, 0.08 | 0.55, 0.06 | t(21) = 0.6 | P = 0.5 |
| PCI (M1) | 0.52, 0.056 | 0.55, 0.04 | t(21) = 1.9 | P = 0.07 |
| PCI (PM) | 0.52, 0.04 | 0.55, 0.06 | t(19) = 1.6 | P = 0.1 |
| LZc EO | 0.84, 0.02 | 0.85, 0.03 | t(21) = 1.5 | P = 0.1 |
| LZc EC * | 0.80, 0.03 | 0.84, 0.05 | t(21) = 3.9 | P = 0.0009 |
| GMFP (S1) | 331.06, 243.59 | 291.31, 129.95 | t(21) = -0.8 | P = 0.5 |
| GMFP (M1) | 267.73, 119.87 | 303.36, 161.84 | t(21) = 1.0 | P = 0.4 |
| GMFP (PM) | 246.69, 133.08 | 221.67, 124.34 | t(19) = -0.8 | P = 0.4 |
| ERSP (S1) | 2.31, 1.12 | 2.63, 1.23 | t(21) = 1.1 | P = 0.28 |
| ERSP (M1) * | 1.97, 0.65 | 2.75, 1.49 | t(21) = 3.0 | P = 0.006 |
| ERSP (PM) | 1.90, 0.96 | 2.09, 1.03 | t(19) = 0.80 | P = 0.4 |
| Delta EO | 2.08, 0.74 | 2.28, 1.32 | t(21) = 0.8 | P = 0.4 |
| Delta EC | 2.63, 1.04 | 2.29, 1.30 | t(21) = -1.0 | P = 0.3 |
| Theta EO * | 0.92, 0.59 | 0.69, 0.35 | t(21) = -3.4 | P = 0.003 |
| Theta EC * | 1.82, 1.68 | 0.81, 0.41 | t(21) = -3.2 | P = 0.005 |
| Alpha EO * | 1.60, 1.34 | 0.79, 0.54 | t(21) = -3.3 | P = 0.003 |
| Alpha EC * | 6.02, 5.48 | 2.77, 3.06 | t(21) = -3.3 | P = 0.003 |
| Beta EO | 0.36, 0.18 | 0.29, 0.15 | t(21) = -2.5 | P = 0.02 |
| Beta EC | 0.53, 0.32 | 0.43, 0.35 | t(21) = -1.2 | P = 0.3 |
| Gamma EO | 0.12, 0.07 | 0.12, 0.09 | t(21) = 0.3 | P = 0.8 |
| Gamma EC | 0.11, 0.07 | 0.21, 0.48 | t(21) = 0.9 | P = 0.4 |

**Table S1. Statistics for TMS-evoked features and resting-state EEG average PSD per frequency band and ocular state, Related to Figures 2 and 3.**
EO: Eyes-open, EC: Eyes-closed. Mean and Standard Deviation (std) values are reported in comma-separated pairs. Two-tailed paired-sample t-test values are displayed as t(df), where df is degrees of freedom, alongside the corresponding P-value. Asterisks indicate measures of a significant change after Bonferroni correction, where n = 5 repeated measures for resting-state PSD measures and n = 4 for LZc, where $\alpha$ = 0.05/n. Frequency bands correspond to those defined in the methods.

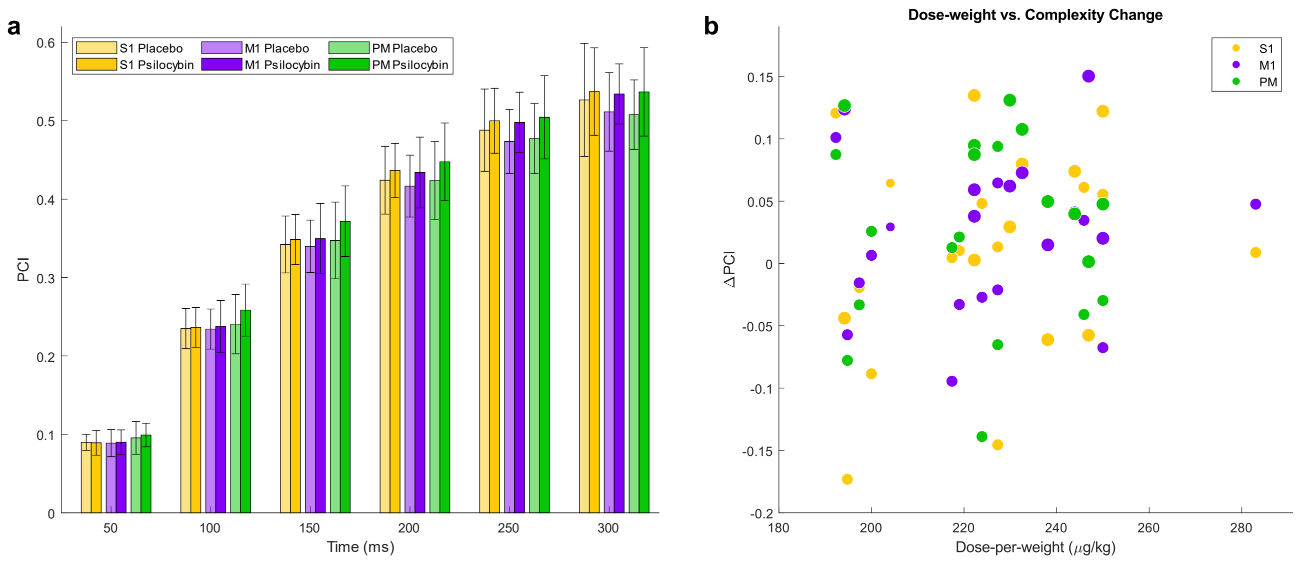


**Figure S1. PCI over time and the effect of dosage, Related to Figure 3.**

**a**) Mean PCI values for 50 ms bins. No significant interactions were found for repeated measures ANOVA within-subject effects of conditions and times for each stimulation site (all of P>0.1). **b**) Change in PCI between conditions as a function of dose-per-weight. Markers are sized proportionally to the absolute dose (10, 15, or 20 mg).

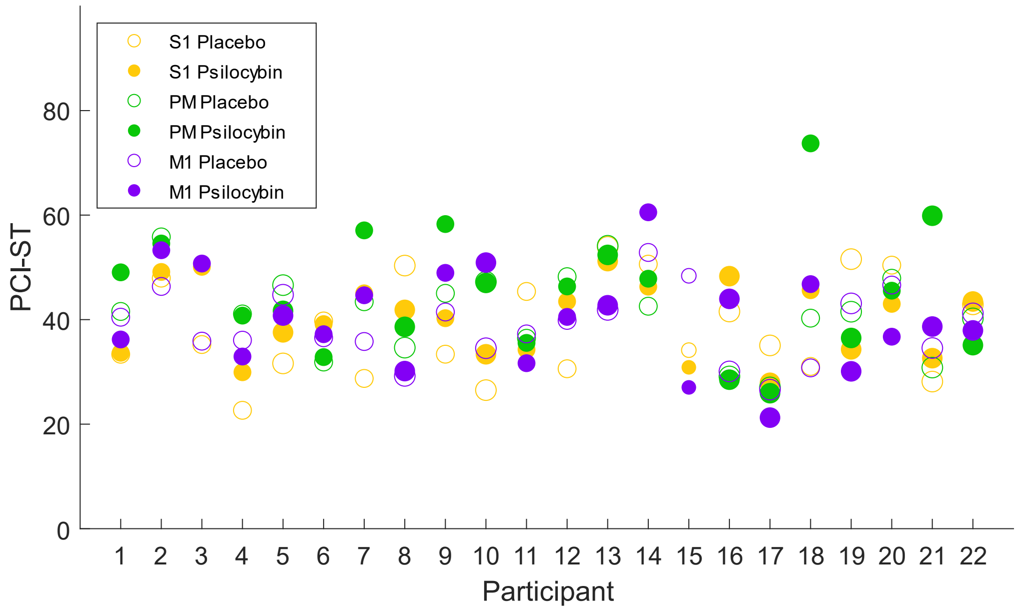


**Figure S2. TMS-evoked complexity measured by PCI-ST, Related to STAR Methods.** PCI-ST values for all participants, color-coded by stimulation site and drug conditions, marker sizes proportional to the psilocybin dose (15 or 20mg). All PCI-ST values satisfy the Single sample Kolmogorov-Smirnov goodness-of-fit hypothesis test for normality and t tests find no statistically significant changes in PCI-ST between conditions. S1: Primary Somatosensory Cortex (yellow, n=22); PM: Premotor Cortex (green, n=20); M1: Primary Motor Cortex (purple, n=22).

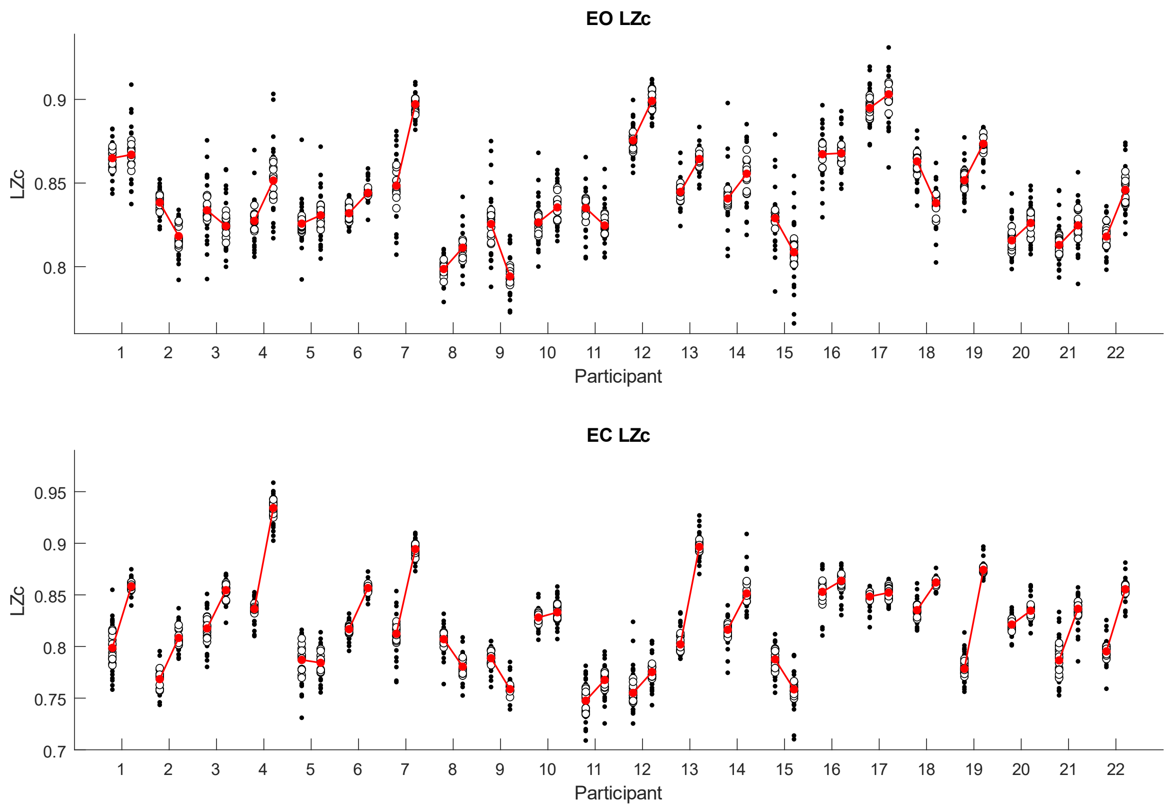


**Figure S3. Individual LZc values are plotted before statistical filtering, Related to STAR Methods.**

(*Top row*) Eyes-open (EO) LZc (y-axis) for individual participants (x-axis) with paired placebo (left) and psilocybin (right) conditions. Each circle is an LZc value for a 10-second non-overlapping period of resting-state EEG activity. White circles are LZc values within the 25-75th percentile for that session and are averaged to provide the LZc value for that session (red circles). Smaller black circles are outliers of the percentile cut-off. Red lines connect the means of each condition to show drug-induced changes. (*Bottom row*) Same format as in the above figure but for the Eyes-closed (EC) condition. Only in the EC condition was there a significant change due to psilocybin (two-tailed paired-sample t-test, $\alpha = 0.05$).

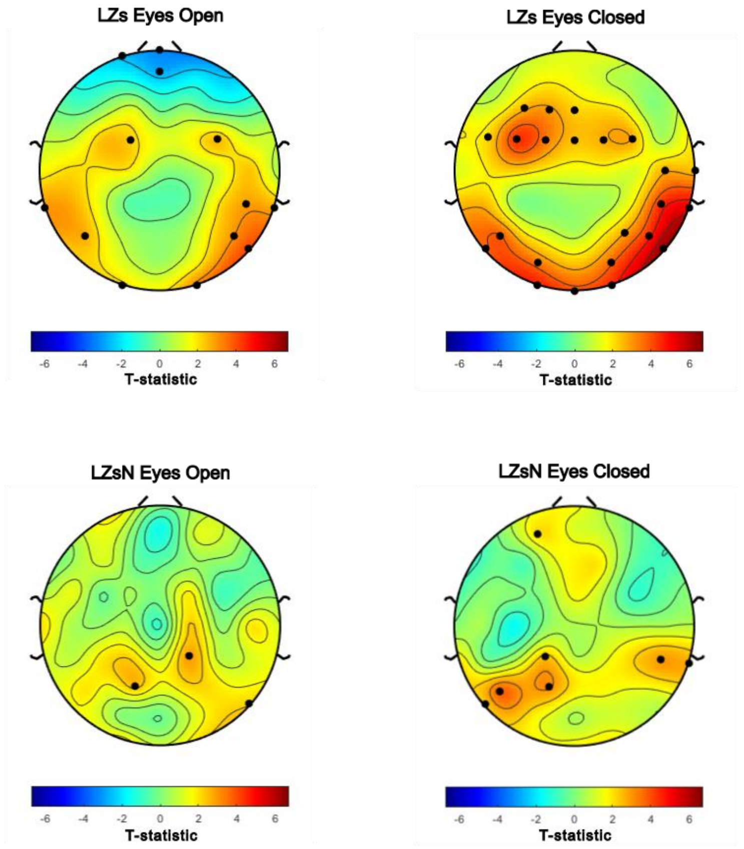


**Figure S4. Single-channel Lempel-Ziv complexity (LZs), Related to STAR Methods.** T-statistic for change in LZs between Psilocybin and Placebo conditions are plotted spatially. LZs values (mean of 10-second epochs for LZ values within 25-75th percentile to avoid outliers) all channels satisfy the Single sample Kolmogorov-Smirnov goodness-of-fit hypothesis test for normality. Paired two-tailed t-tests per channel with correction for false detection rate (Benjamin-Hochberg technique) was applied. Channels with statistically significant T-statistic values ($P<0.05$) after correcting for multiple comparisons are indicated with a black circle. In the second row, the same statistical approach and plotting methods are applied for LZs normalized by phase-shuffling (LZsN) instead of time-shuffled normalization (see Methods).

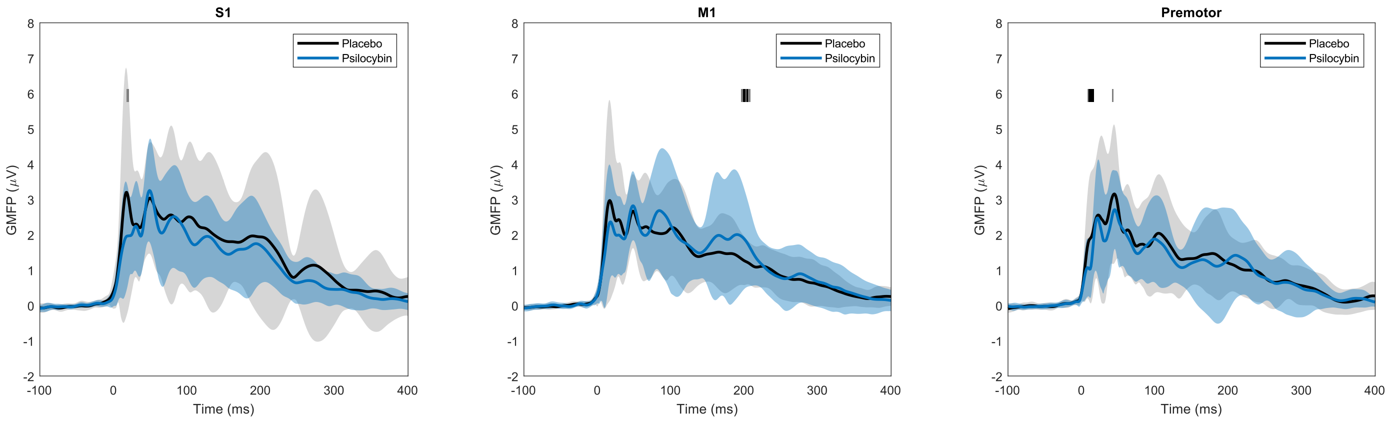


**Figure S5. GMFP per timepoint, Related to Figure 2.**
Mean GMFP (solid curves) is plotted over time with standard deviations overlaid (shaded areas) for placebo (black curve, grey shaded area) and psilocybin (blue curve and shaded area) conditions. All GMFP timepoints for all regions satisfy the Single sample Kolmogorov-Smirnov goodness-of-fit hypothesis test for normality. Paired two-tailed t tests were applied at each timepoint, and significance (P<0.05) is indicated by the presence of a grey marker at that timepoint. The black markers indicate the timepoints where statistical significance is retained after correcting for false detection rate using the Benjamin-Hochberg technique.

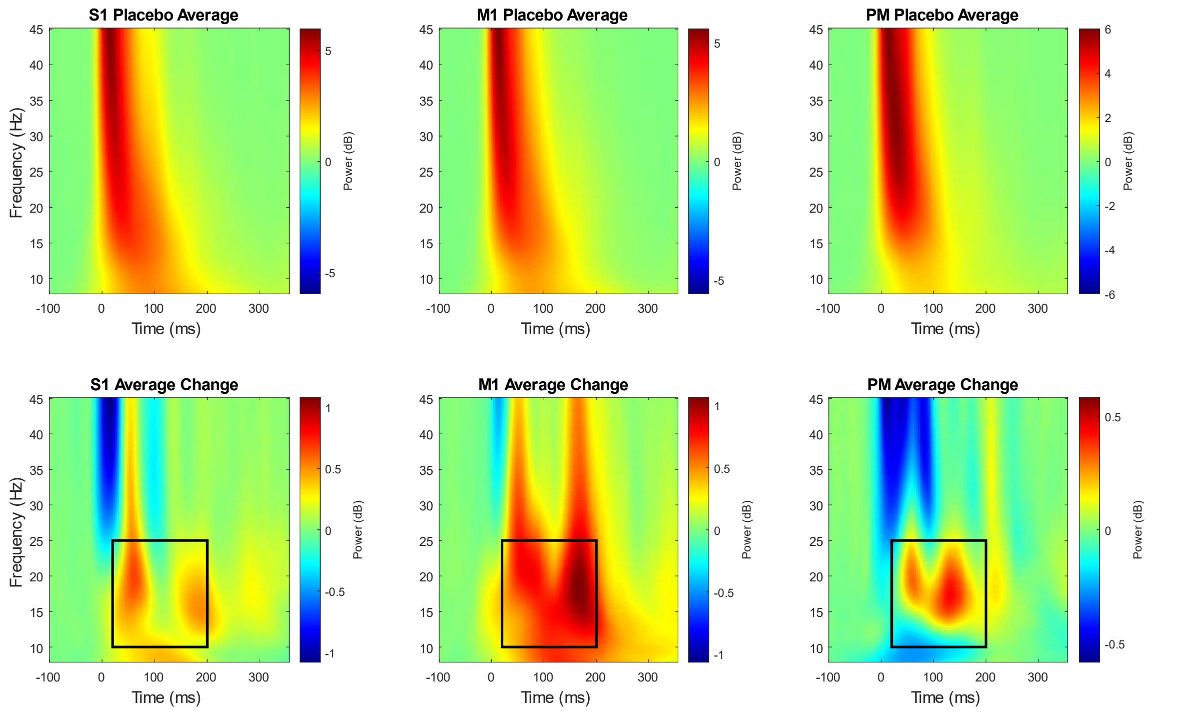


**Figure S6. Spectral analysis of evoked responses to select time and frequency windows of interest, Related to Figure 2.**

The average ERSP plot across all participants (N = 22) for each region during the placebo condition (*top row*). The difference between psilocybin and placebo ERSP plots for each candidate was used to calculate the average effect of psilocybin on the evoked spectra for each stimulation site (*bottom row.* S1: N = 22, M1: N = 22, PM: N = 20). The time-frequency window selected for further analysis (20-200 ms, 10-25 Hz) is outlined by a black rectangle. Colours indicate Power (dB) and symmetrically scaled for the maximum absolute mean intensity across times and frequencies.

| **Dimension** | **Placebo** (mean, std) | **Psilocybin** (mean, std) | **T-value** | **P-value** |
|---|---|---|---|---|
| Elementary Imagery * | 1.65, 5.01 | 63.12, 24.78 | t(21) = 11.5 | $P < 10^{-9}$ |
| Complex Imagery * | 0.76, 1.79 | 67.21, 27.51 | t(21) = 11.1 | $P < 10^{-9}$ |
| Audio-Visual Synaesthesia * | 1.12, 3.29 | 56.44, 34.50 | t(21) = 7.7 | $P < 10^{-6}$ |
| Disembodiment * | 0.56, 0.94 | 44.15, 32.99 | t(21) = 6.2 | $P < 10^{-5}$ |
| Changed Meaning of Percepts * | 1.82, 5.28 | 50.71, 28.75 | t(21) = 8.2 | $P < 10^{-7}$ |
| Cognitive Impairment * | 0.812, 1.15 | 30.94, 19.46 | t(21) = 7.3 | $P < 10^{-6}$ |
| Unity * | 1.35, 4.73 | 53.55, 29.12 | t(21) = 8.8 | $P < 10^{-7}$ |
| Insightfulness * | 0.64, 1.33 | 44.20, 27.65 | t(21) = 7.5 | $P < 10^{-6}$ |
| Blissfulness * | 3.02, 4.6 | 62.58, 30.05 | t(21) = 9.8 | $P < 10^{-8}$ |
| Spiritual experience * | 0.52, 0.99 | 36.56, 29.93 | t(21) = 5.7 | $P < 10^{-4}$ |
| Anxiety | 1.04, 2.60 | 9.33, 14.01 | t(21) = 2.7 | P = 0.01 |

**Table S2. Group-level 11D-ASC statistics, Related to Figure 4.**
Mean and Standard Deviation (std) values are reported in comma-separated pairs. Two-tailed paired-sample t-test values are displayed as t(df), where df is degrees of freedom, alongside the corresponding P-value. Asterisks indicate measures of a significant change after Bonferroni correction, where no. repeated measures = 11.

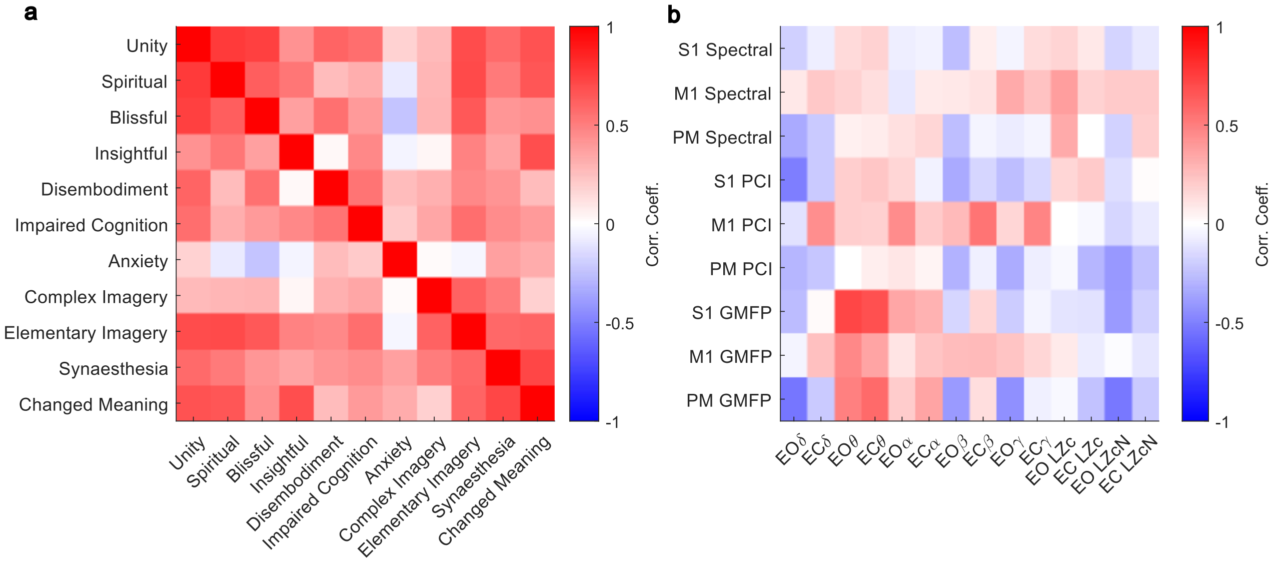


**Figure S7. Correlation matrices for phenomenological and electrophysiological measures, Related to Figures 4.**

**a**) Autocorrelation matrix for change in 11D-ASC scores between conditions (psilocybin – placebo) demonstrate a strong tendency for multicollinearity. **b**) TMS-EEG changes correlated with EEG changes between conditions (psilocybin – placebo). The values displayed are Pearson's correlation coefficients and both figures are scaled by minimum and maximum possible coefficient values, [-1, 1].

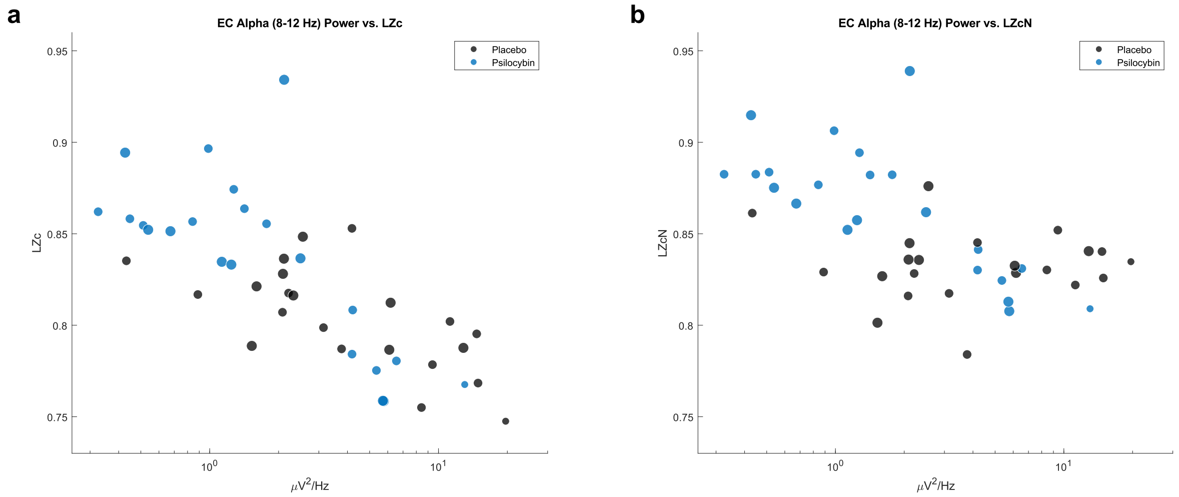


**Figure S8. The relationship between oscillatory power and signal diversity.**

**a**) logarithmic Alpha (8-12 Hz) power is negatively correlated with LZc in both the placebo (black, R = -0.66, P = 0.0009) and psilocybin (blue, R = -0.74, P = 0.0001) conditions during the eyes-closed (EC) state. **b**) Same format as in the left figure but for LZcN. There is also a significant correlation between these log(alpha power) and LZcN in the psilocybin condition (R = -0.73, P = 0.0001) but not for the placebo condition (R = -0.05, P = 0.84).

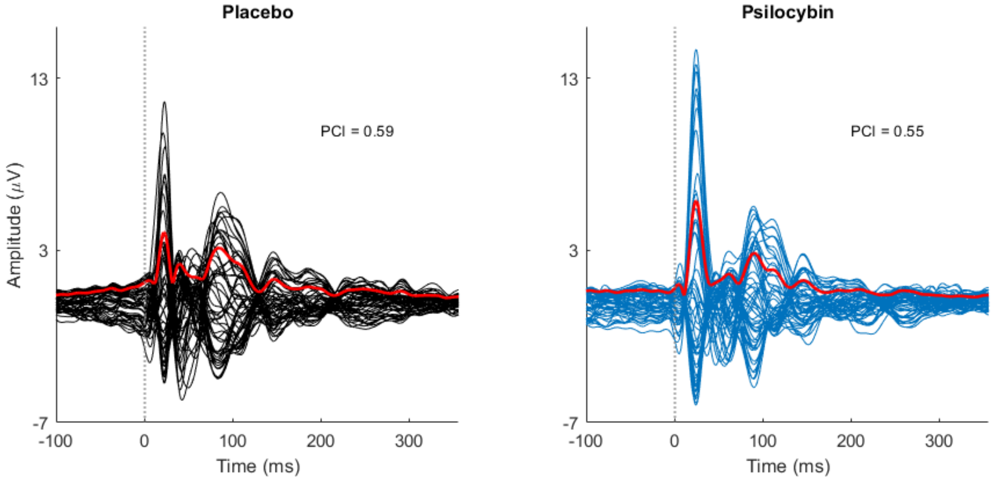


**Figure S9. An example TEP from occipital cortex stimulation, Related to STAR Methods.**
Channel potentials are plotted over time and overlayed to visualize the response for placebo (left, black) and psilocybin (right, blue) conditions. GMFP is overlayed in red and PCI values are displayed above their corresponding TEPs. A neuro-navigation system was not used during these recordings; the lack of guaranteed reproducibility meant that occipital cortex stimulation data were not includible in the main findings.

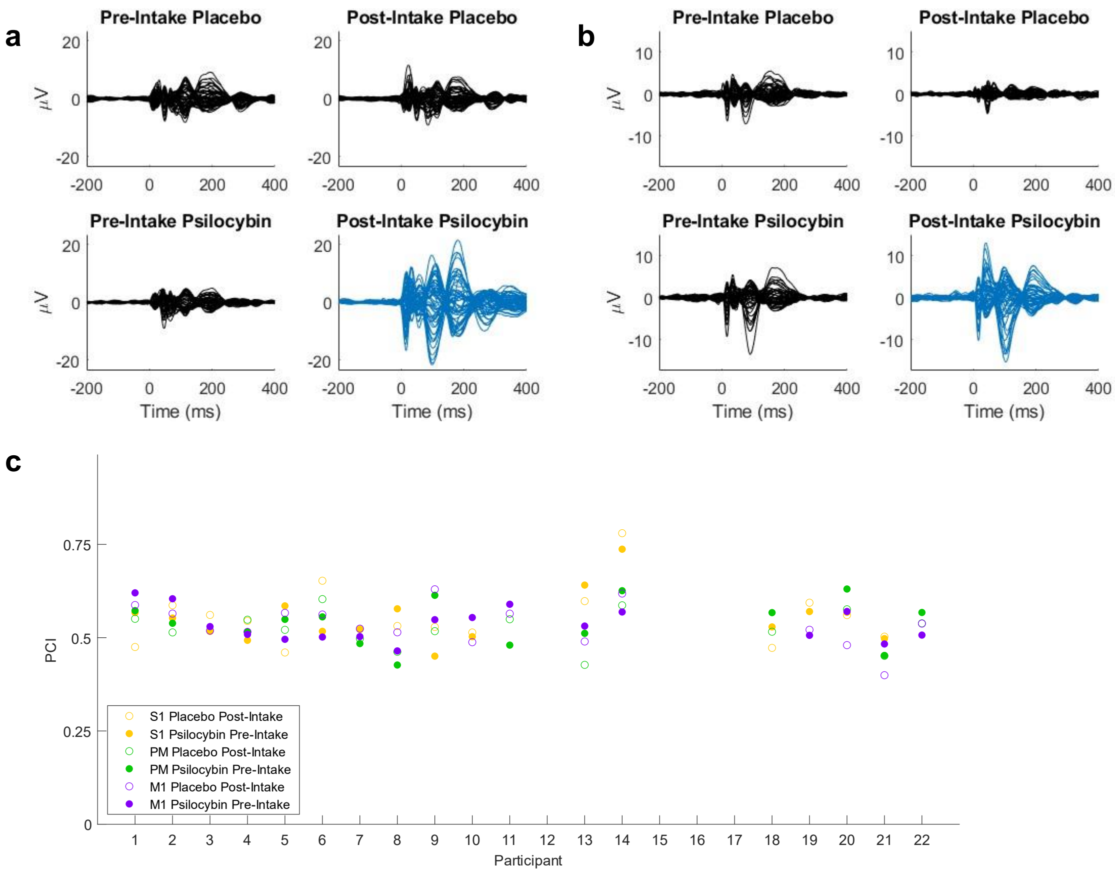


**Figure S10. Alternative non-psilocybin TMS-EEG responses substituted for placebo responses, Related to STAR Methods.**

**a**) An example participant's TMS-EEG responses to M1 stimulation before and after intake of either placebo or psilocybin. Non-psilocybin conditions are reproducible across recording sessions and are therefore stable. **b**) An example participant's TMS-EEG responses (as in (a)) to S1 stimulation, whose placebo recording intended for comparison with psilocybin ('pre-intake placebo') displayed insufficiently low signal strength for meaningful analysis. Both other non-psilocybin ('pre-intake') recordings are comparable (2 weeks apart). In this instance, pre-intake psilocybin was used to substitute this participant's placebo recording for subsequent analyses. **c**) PCI values for all participants, comparing their pre-intake psilocybin and post-intake placebo responses. One-way ANOVA finds no statistically significant changes between conditions and stimulation sites. Data for participants whose pre-intake psilocybin condition recordings were used in the main analysis (due to invalid post-intake placebo recordings) are excluded from this figure.

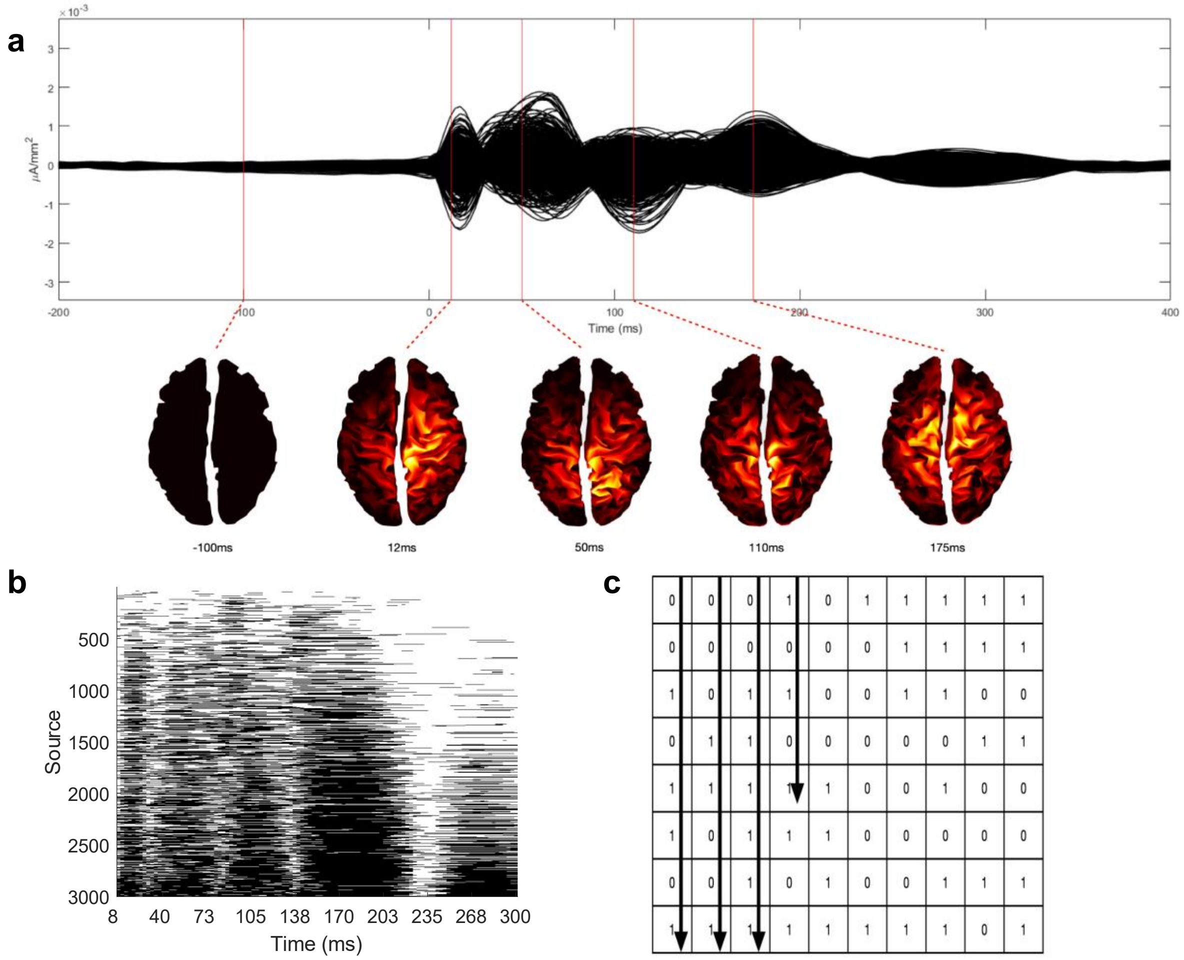


**Figure S11. Summarised procedure for calculating PCI, Related to STAR Methods.**
**a**) TMS-evoked responses at the sensor level (electrode voltages) were used for source localization. Electrode activity was used to infer the current source activity of 3000 cortical points and their activity is plotted over time. Below, time points of interest are shown as projected activity on the cortical surface of a template cortical mesh (filtered for $p < 0.01$ significance relative to –100 to 0 ms baseline activity). **b**) Non-parametric statistics were applied to threshold the responses at the single-source level (0's = non-significant, 1's = significant, relative to the baseline period). Sources are sorted by their total significant events. **c**) Lempel-Ziv complexity was calculated by cataloguing unique sequences of binary strings in single time-step partitions. The total number of unique binary strings in the matrix shown in (b) is then the complexity of that TMS-evoked response. This value of complexity is then normalized to the binary entropy of the matrix to produce the Perturbational Complexity Index (PCI) for that response. The method was replicated from [1].

## References

1. Casali, A.G., Gosseries, O., Rosanova, M., Boly, M., Sarasso, S., Casali, K.R., Casarotto, S., Bruno, M.A., Laureys, S., Tononi, G., and Massimini, M. (2013). A theoretically based index of consciousness independent of sensory processing and behavior. Sci Transl Med *5*, 198ra105. 10.1126/scitranslmed.3006294.
